# Comparison of linear motion perception thresholds in vestibular migraine and Menière’s disease

**DOI:** 10.1007/s00405-015-3835-y

**Published:** 2016-01-04

**Authors:** Tatiana Bremova, Arla Caushaj, Matthias Ertl, Ralf Strobl, Nicolina Böttcher, Michael Strupp, Paul R. MacNeilage

**Affiliations:** 1 German Center for Vertigo and Balance Disorders, University Hospital Munich, Marchioninistrasse 15, 81377 Munich, Germany; 2 Department of Neurology, University Hospital Munich, Marchioninistrasse 15, 81377 Munich, Germany; 3 Institute for Clinical Neurosciences, University Hospital Munich, Marchioninistrasse 15, 81377 Munich, Germany; 4 Graduate School of Systemic Neurosciences, Ludwig-Maximilians University Munich, Grosshaderner Strasse 2, 82152 Munich, Germany; 5 Institute for Medical Information Processing, Biometrics and Epidemiology, Ludwig-Maximilians University Munich, Marchioninistrasse 17, 81377 Munich, Germany

**Keywords:** Perception thresholds, Vestibular migraine, Menière’s disease, Otolith function, Utricle, Saccule, Diagnostic accuracy

## Abstract

Linear motion perceptual thresholds (PTs) were compared between patients with Menière’s disease (MD) and vestibular migraine (VM). Twenty patients with VM, 27 patients with MD and 34 healthy controls (HC) were examined. PTs for linear motion along the inter-aural (IA), naso-occipital axes (NO), and head-vertical (HV) axis were measured using a multi-axis motion platform. Ocular and cervical vestibular evoked myogenic potentials (o/c VEMP) were performed and the dizziness handicap inventory (DHI) administered. In order to discriminate between VM and MD, we also evaluated the diagnostic accuracy of applied methods. PTs depended significantly on the group tested (VM, MD and HC), as revealed by ANCOVA with group as the factor and age as the covariate. This was true for all motion axes (IA, HV and NO). Thresholds were highest for MD patients, significantly higher than for all other groups for all motion axes, except for the IA axis when compared with HC group suggesting decreased otolith sensitivity in MD patients. VM patients had thresholds that were not different from those of HC, but were significantly lower than those of the MD group for all motion axes. The cVEMP p13 latencies differed significantly across groups being lowest in VM. There was a statistically significant association between HV and NO thresholds and cVEMP PP amplitudes. Diagnostic accuracy was highest for the IA axis, followed by cVEMP PP amplitudes, NO and HV axes. To conclude, patients with MD had significantly higher linear motion perception thresholds compared to patients with VM and controls. Except for reduced cVEMP latency, there were no differences in c/oVEMP between MD, VM and controls.

## Introduction

Vestibular migraine (VM) is a frequent vestibular syndrome characterized by recurrent vertigo attacks of moderate or severe intensity in association with aura and migrainous headaches [1, 2]. This can be difficult to differentiate from Menière’s disease (MD), which is also characterized by episodic vertigo with additional fluctuating hearing loss, aural fullness and tinnitus [3]. A correct diagnosis is crucial for a successful treatment, as patients suffering acute vertigo attacks are neither capable to work nor to participate in daily activities such as driving a car [4]. Even the demonstration of endolymphatic hydrops (EH) by locally enhanced inner ear imaging (LEIM) [5] does not discriminate because EH has been found in both diseases [6]. In addition, patients with MD often develop migrainous headaches, and vice versa, patients with VM can suffer from hearing problems [7, 8]. These findings imply that there is a significant clinical overlap between VM and MD with a need for additional testing to differentiate between them.

A well-known phenomenon in VM is motion hypersensitivity accompanied by motion sickness [9–11]. We therefore hypothesized that perceptual thresholds (PTs), defined as the stimulus magnitude at which subjects can first perceive the motion direction despite the noise inherent in sensory transduction and subsequent neural processing [12], would be reduced in VM [13]. PT testing might therefore be helpful for VM–MD differentiation. In fact, reduced PTs in VM compared to healthy controls have been observed previously [13], but only during mid-frequency roll rotation while upright, which stimulates both otoliths and canals. In contrast, here we test linear motion thresholds, which should rely predominantly on otolith function. We expected linear motion thresholds to be elevated in MD because of previous reports that MD negatively impacts otolith function [14, 15]. We also investigated otolith function by ocular and cervical vestibular evoked myogenic potentials (c/oVEMP). Further, the dizziness handicap inventory (DHI) was applied to assess the individual functional impairment of patients depending on diagnosis, motion thresholds and VEMP. These methods are similar to those we have used previously [16], but here we focus specifically on the problem of differentiating VM from MD. Diagnostic accuracy of all applied methods for discriminating between VM and MD pathologies is also assessed and compared.

## Subjects and methods

### Subjects

Patients were recruited from the interdisciplinary outpatient clinic. The study included 20 patients with definite or probable VM [11 females (F), mean age 40.9 years] according to the recent diagnostic criteria [2], 27 patients with clinically probable or definite MD (13 F, 58 years), according to the recent current criteria [3], as well as a group of 34 healthy subjects with no prior history of dizziness, neurologic or neurootologic disease (21 F, 44.6 years). All patients had a standardized neurologic and neuro-ophthalmologic examination, including video-oculography with caloric irrigation. All patients with VM had normal caloric irrigation testing, as defined by the mean peak slow phase velocity (mPSPV) of vestibular nystagmus >5°/s, whereas patients with MD had a lowered canal excitability with the mPSPV of <5°/s on the affected ear. The study was performed in accordance with the Helsinki II Declaration and approved by the ethics committee of the Ludwig-Maximilians University Medical Faculty. All participants gave their informed consent prior to their inclusion in the study.

### Recording of ocular VEMP

Examination was performed in the supine position with subjects’ upper bodies at a 30° angle from the horizontal. To ensure maximum upgaze was maintained subjects fixated a small target at the mini-shaker margin, i.e. superomedial gaze of approximately 30°. This angle is reported to elicit the largest responses [17, 18]. “Mini tap” stimuli were administered with a Bruel and Kjaer Mini-Shaker Type 4810 (2-ms clicks positive polarity at 2 Hz) at the Fz cranial site (in the midline at the hairline, 30 % of the distance between the inion and nasion). These taps generate an acceleration wave that propagates to the mastoid bilaterally, leading to an outward linear stimulation of the utricles. A cleaning and de-greasing procedure was performed with abrasive paste before recording. The recording electrode was placed over the contralateral inferior oblique muscle (centered beneath the pupil and 3 mm below the eye), the reference electrode was placed on the chin, and a ground electrode was placed under the chin. Responses were averaged over 50–100 stimuli. n10 and p15 were identified as the first negative and positive peaks that occurred between 10 and 20 ms after stimulus onset, respectively. Responses were amplified by a Bruel and Kjaer Type 2718 power amplifier (voltage gain 30 dB). Unrectified signals were averaged with filter cutoffs of 20–500 Hz. n10 amplitude [19] and latency were taken as dependent measures. These methods are very similar to those used previously [16].

### Recording of cervical VEMP

Examination was performed in the same supine position for cVEMPs. In addition, subjects were instructed to lift the head to generate the active neck flexion that is needed during cVEMP and recording of tonic background muscle activity. Tone bursts were played monaurally via intro-aurical speakers with foam ear-tips (Air-conducted 500-Hz, 125-dB SPL). A recording electrode was located at the belly of the ipsilateral sternocleidomastoid muscle, a reference electrode was placed on the manubrium sterni, and a ground electrode was placed on the forehead. EMG responses to 50–100 stimuli were averaged after activity was amplified and bandpass filtered (Nicolet Biomedical Inc, Madison WI, USA). p13 and n23 were defined as the first positive and negative peaks occurring between 13 and 23 ms after stimulus onset, respectively. The p13 latency and the corrected peak-to-peak amplitude (PP) were taken as the dependent measures; this amplitude is defined as the difference between the p13 and n23 peaks. Correction was performed by taking the ratio of PP amplitude divided by the mean EMG activity over the recording period [20]. These methods are very similar to those used previously [16].

### Linear motion perception threshold testing

To measure perceptual thresholds, subjects were physically moved while seated on a hexapod motion platform (Moog 6DOF2000E). They sat in a padded racing seat and wore a 5-point harness. The head was cradled in a form-fitted vacuum pillow and secured with a forehead strap. To cancel and mask the sound of the moving platform, white noise was played through noise cancellation headphones. To eliminate visual cues, blackout goggles were worn. A wireless numeric keypad was used to collect responses. On each trial, a 1-s linear movement was presented in one of two opposite directions and subjects indicated the direction that they had moved, a two-alternative-forced-choice task. A raised cosine velocity profile with frequency 1 Hz was presented. Axis of movement depended on the condition, either (1) left or right along the inter-aural (IA) axis, (2) forward or backward along the naso-occipital (NO) axis, or (3) up or down along the head-vertical (HV) axis. Each condition was run in a separate block; blocks were run in a random order for each subject.

To assess threshold, movement magnitude was varied from trial to trial using a staircase procedure. The largest stimulus was a 15 cm displacement (peak acceleration, 94.25 cm/s^2^); displacement was decreased by one-third with every step down on the staircase (15, 10, 6.66 cm, etc.). Duration was fixed at 1-s, so displacement, velocity, and acceleration scaled together. Each block started with the largest displacement (15 cm). Achieved acceleration was not measured but in previous work, we have verified that the platform reproduces the desired trajectories very accurately [21].

**Fig. 1:**
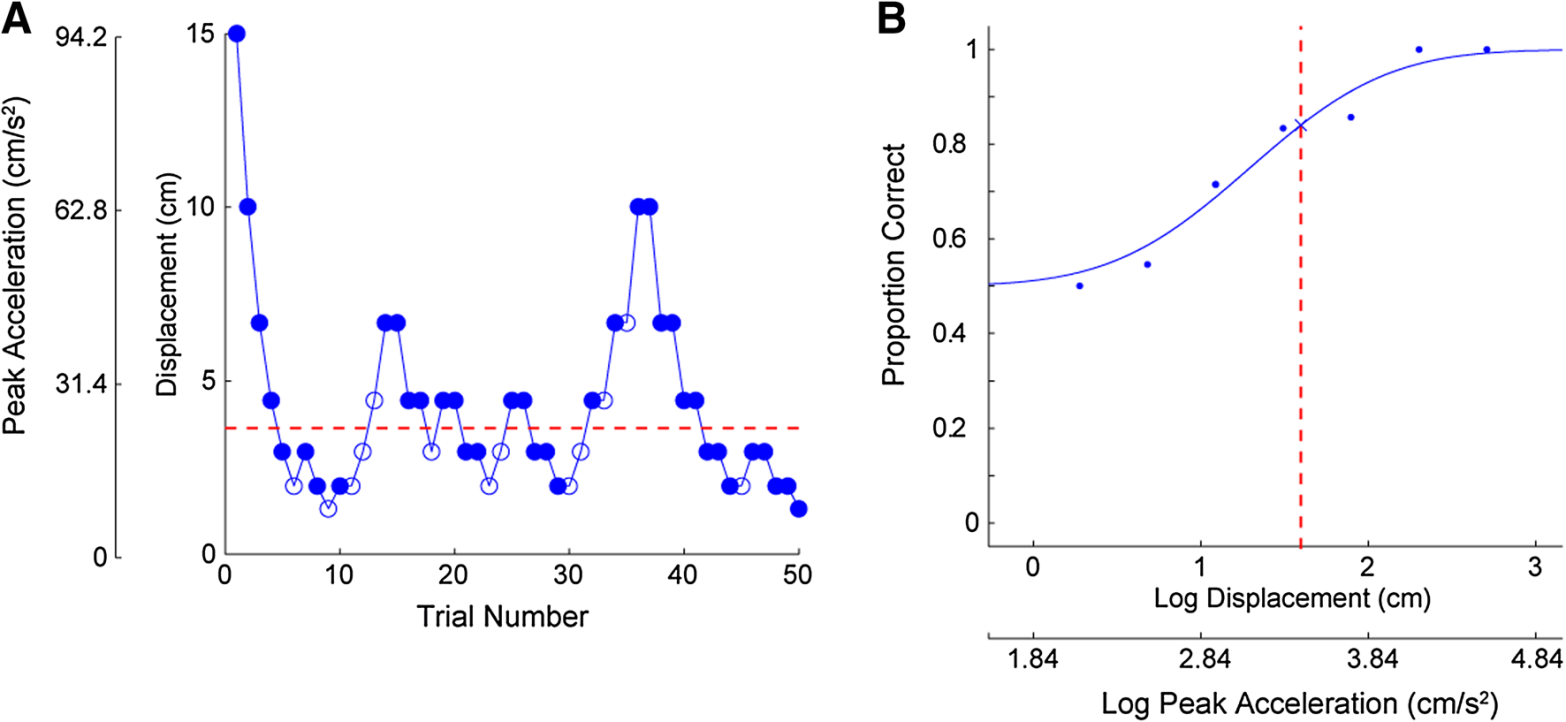
Example of individual staircase history (**a**) and psychometric fit (**b**). The 2D1U staircase terminated after 50 trials. *Filled and unfilled points* show correct and incorrect responses, respectively. **b** Cumulative Gaussian fit to data from (**a**). Proportion of correct responses is plotted as a function of the log of the stimulus magnitude. Threshold is the stimulus value corresponding to 84 % correct shown by *red dashed lines* in **a** and **b**

The staircase began using a 1-up-1-down stepping rule. Stimulus magnitude was decreased after a correct answer and increased after an incorrect answer. This allowed the staircase to converge quickly to smaller magnitudes where performance was close to chance level. The staircase rule was changed after four reversals (i.e., a step down followed by a step up or a step up followed by a step down). For remaining block a 2-down-1-up (2D1U) stepping rule was used. Stimulus magnitude was reduced after two consecutive correct answers, and increased after each incorrect answer. A total of fifty trials were performed, and this required ~8 min/axis (Fig. 1a). Several prior studies of vestibular perception have used similar methods [22–27]. A psychometric function was fit to the data from each block using a maximum likelihood method [28, 29] to find the stimulus magnitude that results in 84 % correct performance, i.e., one standard deviation from chance (50 %) performance (Fig. 1b). This quantifies the standard deviation of the noise on the perceptual self-motion estimate [24, 30]. Because the range of motion of the platform is limited, thresholds could not be reliably measured above an upper limit.Thus, threshold was assigned equal to the largest stimulus magnitude (94.25 cm/s^2^) where performance was <84 % correct at the largest stimulus. These methods are very similar to those used previously [16].

### Dizziness handicap inventory

To quantitatively assess the self-perceived impairment in daily life, subjects were administered the dizziness handicap inventory (DHI). The DHI is a 25-item questionnaire developed for quantifying the impact of dizziness on everyday life. It is divided in three parts: physical, functional and emotional. Each question may be answered as “yes”, “sometimes” and “no” with a “yes” response yielding a score of 4, “sometimes” response a score of 2 and “no” a score of 0. The overall as well as subscale (physical, functional and emotional) scores were computed (DHI_T, DHI_P, DHI_F, DHI_E). The test version, as published in 1990 [31], was used. The DHI was set to 0 in healthy controls, as they had no prior history of dizziness, neurologic or neurootologic disease.

### Statistical analysis

Analysis and graph design was performed using SPSS version 22.0 (IBM, New York, NY, USA). A univariate analysis of covariance (ANCOVA) was run to determine if there were mean differences in vestibular thresholds, oVEMP and cVEMP amplitude and latencies, and DHI between three study groups (VM, MD, HC) after controlling for age. Post hoc analysis was performed with a Bonferroni adjustment. Outliers were excluded; these were defined as values outside the mean ± 2SD for a particular axis and group. Spearman’s rank correlation coefficients were computed to compare vestibular physiological test measures and age in controls. Receiver operating characteristic curve (ROC), with VM representing a positive actual state and MD a negative one, was applied to visualize the potential quantitative cut-points for differentiating VM from MD and to find out the tradeoff between sensitivity and specificity of performed measures. Classification performance of each measure was described using the area under the curve (AUC). In order to identify the optimal cut-point we used the Youden Index (*J*); the Youden index is a function of the sensitivity (*q*) and the specificity (*p*) of a classifier and is defined as: $J = q + p - 1$. The optimal cut-point was defined as the one maximizing *J*.

## Results

### Linear motion thresholds

**Fig. 2:**
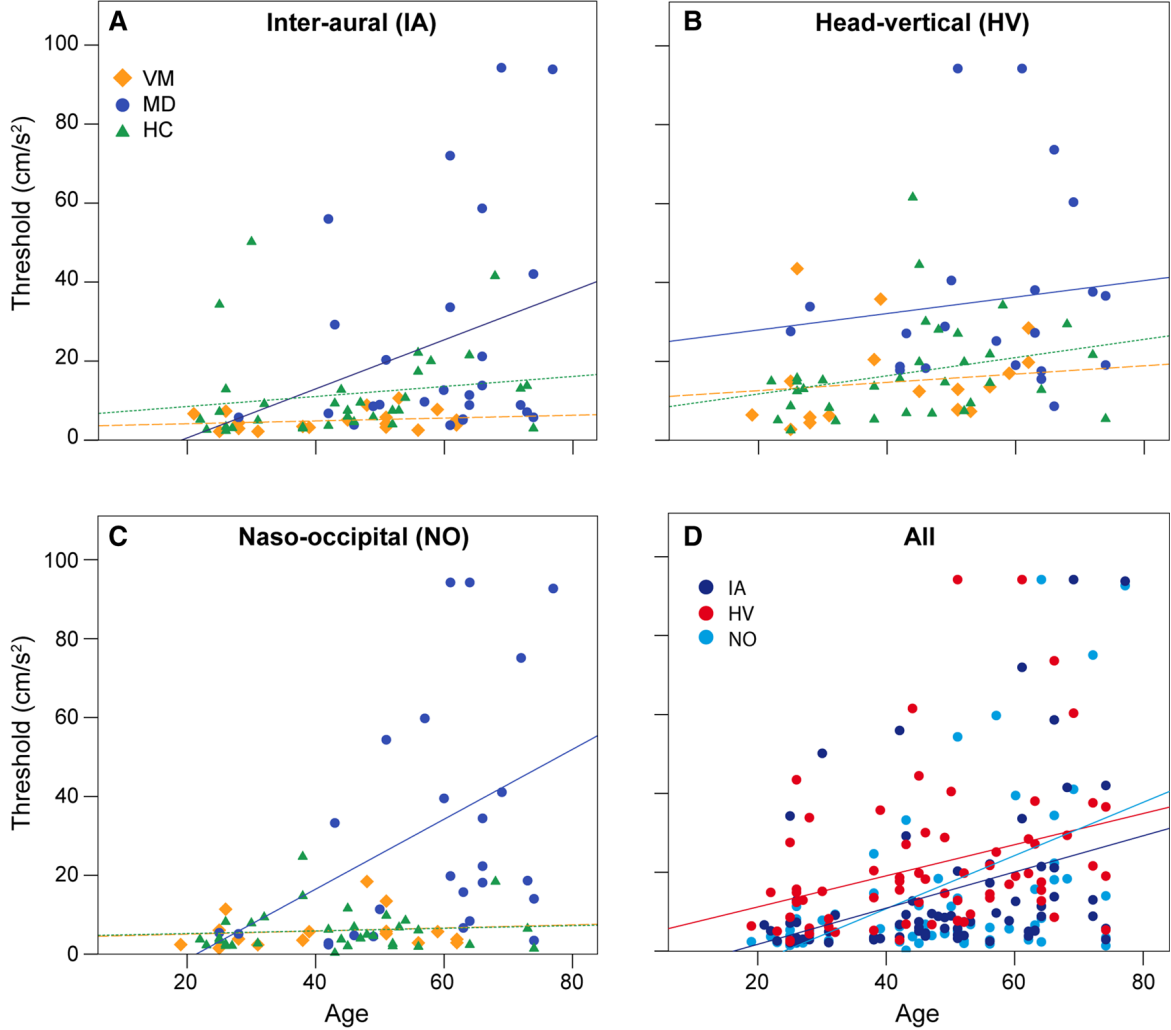
Vestibular perceptual thresholds (PTs). **a** Inter-aural (IA) axis, **b** Head-vertical (HV) axis, **c** Naso-occipital (NO) axis. *Lines* show linear fits by study group. Vestibular migraine (VM) indicated by *yellow diamonds* and *dashed line*. Menière’s disease (MD) indicated by *blue circles* and *solid line*. Healthy controls (HC) indicated by *green triangles* and *dotted line*. **d** Thresholds replotted for all study groups to compare axes: IA, HV, and NO, indicated by *dark blue*, *red*, and *light blue circles* and *lines*, respectively

**Fig. 3:**
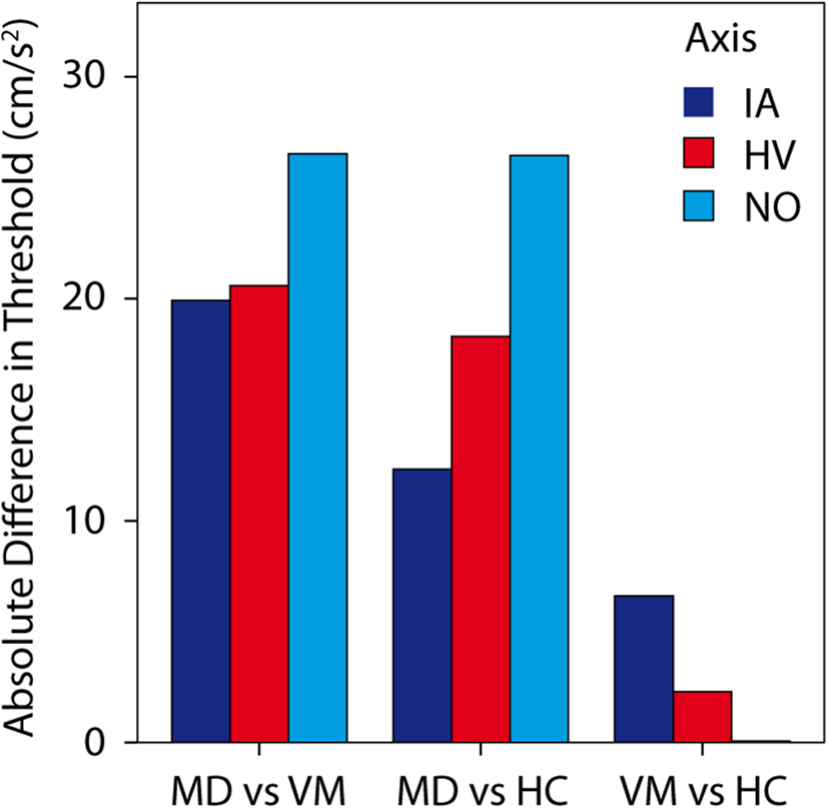
Absolute difference (*delta*) in perceptual thresholds between groups; Menière’s disease (MD), vestibular migraine (VM), heathy controls (HC). *Statistically significant values on the level of *p* < 0.05. **Statistically significant values on the level of *p* < 0.001

Threshold data by group and axis are displayed in Fig. 2a–c, and mean threshold values are listed in Table 1. After adjustment for age, there was a statistically significant difference in perceptual thresholds between the groups (IA: *F* = 3.387, *p* = 0.025; HV: *F* = 6.595, *p* = 0.002; NO: *F* = 8.844, *p* = 0.000368) as revealed by ANCOVA performed for each motion axis separately. Highest thresholds were observed for the MD group, and post hoc tests among the groups (Table 2) revealed that these thresholds were significantly higher than those of the other groups for all motion axes, except for IA axis when compared with HC group (Fig. 3). In contrast, the lowest thresholds were observed for the VM group; these thresholds were significantly lower than for the MD group for all motion axes. When comparing thresholds across axes regardless of subject group (Fig. 2d), there was a trend for HV thresholds to be higher than thresholds for the other axes (*F* = 2.954, *p* = 0.054), being highest in the MD group (Table 2). Perceptual thresholds between groups were subtracted to obtain delta (Δ) values. The highest values were observed for comparison of VM and MD groups along the NO axis (Fig. 3). Linear motion perception thresholds were significantly correlated with age (*p* < 0.01), as tested by Spearman correlation analysis (IA: *r* = 0.384, HV: *r* = 0.341, NO: *r* = 0.445).

**Table 1:** Mean (SD) linear motion perceptual thresholds, ocular and corrected cervical vestibular evoked myogenic potentials (VEMP) amplitudes and latencies and dizziness handicap inventory (DHI) scores in vestibular migraine (VM), Menière’s disease (MD) and healthy control (HC) groups

Patient group	*N*	Age	N10 oVEMP amplitudes (µV)	N10 oVEMP latencies (ms)	Corrected PP cVEMP amplitude (μV)	P13 cVEMP latencies (ms)	Threshold IA^a^ (cm/s^2^)	Threshold HV^b^ (cm/s^2^)	Threshold NO^c ^(cm/s^2^)	DHI total
Vestibular migraine	20	40.9 (14.5)	12.61 (7.52)	9.9 (2.1)	0.95 (0.35)	14.8 (3.1)	4.98 (2.42)	14.85 (11.26)	5.94 (4.62)	38
Menière’s disease	27	58 (13.4)	10.35 (8.53)	9.7 (1.3)	0.68 (0.68)	17.6 (4.1)	24.91 (27.61)	35.44 (24.15)	32.47 (32.15)	36.3
Healthy controls	34	44.6 (15.2)	10.75 (4.77)	10.5 (2.7)	1.76 (4.1)	17.1 (3.3)	11.59 (11.4)	17.14 (12.52)	6.01 (5.23)	0

*N* number of patients, *DHI* dizziness handicap inventory, *VEMP* vestibular evoked myogenic potentials, *IA* inter-aural, *HV* head-vertical, *NO* naso-occipital axes

^a^For the analysis of IA perceptual thresholds, three outliers (one VM, two HC) were excluded

^b^For the analysis of HV perceptual thresholds, four outliers (two MD, two HC) were excluded

^c^For the analysis of NO perceptual thresholds, two outliers (one VM, one HC) were excluded

**Table 2:** Mean (SD) differences in linear motion perception thresholds and associated *p*-values for comparison between Menière’s disease (MD), vestibular migraine (VM) and healthy control groups (HC) for each axis

	ΔIA	IA *p*-value	ΔHV	HV *p*-value	ΔNO	NO *p*-value
MD vs VM	19.93 (25.19)	0.022*	20.59 (12.89)	0.007*	26.53 (27.53)	0.005*
MD vs HC	12.32 (16.21)	0.17	18.3 (11.63)	0.005*	26.46 (26.92)	<0.001**
VM vs HC	6.61 (8.98)	0.672	2.29 (1.26)	1	0.07 (0.61)	1

Δ absolute difference between perceptual thresholds in examined groups

*IA* Inter-aural,* HV* head-vertical,* NO* naso-occipital (NO) axes

* Statistically significant values at the level of *p* < 0.05

** Statistically significant values at the level of *p* < 0.001

### Discrimination between VM and MD

An overview of the ROC analysis for each measure is shown in Table 3. The most robust measure according to the AUC was the IA threshold (AUC = 0.848, 95 % CI 0.737–0.959, *p* = *p < *0.001), followed by the cVEMP PP amplitudes (AUC = 0.796, 95 % CI 0.647–0.944, *p* = 0.001), NO axis (AUC = 0.789, 95 % CI 0.657–0.921, *p* = 0.001) and HV axis (AUC = 0.775, 95 % CI 0.628–0.923, *p* = 0.002). Ocular VEMP amplitudes and latencies and DHI questionnaire were not diagnostically relevant.

**Table 3:** Characteristics of the diagnostic accuracy of the linear motion perceptual thresholds, ocular and corrected cervical vestibular evoked myogenic potentials (VEMP) amplitudes and latencies and dizziness handicap inventory (DHI) scores in vestibular migraine (VM), Menière’s disease (MD) and healthy control (HC) groups

	N10 oVEMP amplitudes	N10 oVEMP latencies	Corrected PP cVEMP amplitudes	P13 cVEMP latencies	Threshold IA	Threshold HV	Threshold NO	DHI total
AUC	0.609	0.461	0.796	0.71	0.848	0.775	0.789	0.442
*p* value	0.212	0.655	0.001	0.02	0.001	0.002	0.001	0.515
95 % confidence interval	0.445–0.773	0.272–0.650	0.647–0.944	0.554–0.866	0.737–0.959	0.628–0.923	0.657–0.921	0.267–0.617
Sensitivity	68 %	63 %	83 %	72 %	84 %	72 %	88 %	63 %
Specificity	44 %	22 %	80 %	64 %	70 %	83 %	63 %	32 %
Cut-point	7.94	10.33	0.62	16.75	8.17	17.26	13.75	43

*AUC* area under curve, *DHI* dizziness handicap inventory, *VEMP* vestibular evoked myogenic potentials, *IA* inter-aural, *HV* head-vertical, *NO* naso-occipital axes

### Ocular and cervical VEMP testing

Mean o/cVEMP values are listed in Table 1. The cVEMP PP amplitudes did not significantly differ among the study groups (*F* = 0.972, *p* = 0.383), but the cVEMP p13 latencies tended to be reduced in VM (*F* = 3.104; *p* = 0.040; post hoc: VM vs. MD group: *p* = 0.052, VM vs. HC group: *p* = 0.097, MD vs. HC group: *p* = 1.0). Across groups there was a statistically significant association between thresholds and cVEMP PP amplitudes for HV and NO axes, but this association did not reach significance for the IA axis (IA-cVEMP: *r* = −0.175, *p* = 0.139; HV-cVEMP: *r* = −0.254; *p* = 0.034; NO-cVEMP: *r* = −0.251; *p* = 0.034).

The oVEMP amplitudes and latency showed no significant differences or trends based on ANCOVA across the patient groups (n10-latency: *F* = 0.670, *p* = 0.514; n10 amplitudes: *F* = 0.355, *p* = 0.702). Across groups there were no significant associations between thresholds and oVEMP n10 amplitudes (IA-oVEMP: *r* = 0.096, *p* = 0.402; HV-oVEMP: *r* = 0.146, *p* = 0.207; NO-oVEMP: *r* = 0.028, *p* = 0.812).

### Dizziness handicap inventory

DHI, a measure of the subjective impairment, did not differ between analyzed groups (DHI_T: *F* = 0.010, *p* = 0.919; DHI_P: *F* = 0.34, *p* = 0.855; DHI_F: *F* = 0.75, *p* = 0.785; DHI_E: *F* = 0.11, *p* = 0.917). There was no statistically significant relationship between DHI and thresholds (DHI-IA: *r* = −0.047, *p* = 0.770; DHI-HV: *r* = −0.054, *p* = 0.742; DHI-NO: *r* = −0.152, *p* = 0.342) or o-/cVEMP n10/PP amplitudes (oVEMP: *r* = −0.163, *p* = 0.155; cVEMP: *r* = 0.136, *p* = 0.403).

## Discussion

The major findings of this study were as follows: first, thresholds were significantly elevated in patients with MD, as expected due to underlying vestibular deficits. This difference was most observable for HV thresholds, which reinforces the evidence of predominantly saccular dysfunction in these patients [5, 32]. Second, thresholds were lowest in VM, but not significantly lower than thresholds measured in HC. This finding supports the hypothesis that VM does not result from a general increased vestibular sensitivity. Instead, as previously suggested, increased vestibular sensitivity in VM could be a manifestation of abnormal central integration of canal and otolith signals, perhaps at the level of the caudal cerebellar vermis [33]. Other mechanisms such as increased excitability in thalamus [34] or alteration of brain regions characteristic for pain, multisensory vestibular processing and central vestibular compensation [35] in patients suffering VM may also play a role. Finally, analysis of the diagnostic accuracy revealed that thresholds are a good clinical tool to discriminate between VM and MD patients.

Perceptual thresholds allow for the measurement of perceptual function and separate testing of linear and angular motion sensitivity along or around different axes, which allows independent assessment of particular vestibular organs (i.e., utricle, saccule, and horizontal and vertical canals [16, 30, 36, 37]). For these reasons, PTs have the potential to become a highly relevant clinical diagnostic method in future.

At present, the standard method to assess otolith function in the clinic is using VEMP. Therefore, we also assessed whether VEMP allow differentiation between study groups, and whether VEMP are correlated with PTs. Prior studies have reported that amplitudes of cVEMP, thought to reflect saccular function, are reduced in VM [38] and MD [39] compared to healthy controls, but we found no significant differences among groups. Nevertheless, the correlation between cVEMP PP amplitudes and PTs was significant for HV and NO axes, both of which rely partly (NO) or predominantly (HV) on saccular function, since the sacculus is oriented approximately in the sagittal plane of the head. While this correlation was not observed in our previous study [16], we nevertheless interpret this as an indication that both cVEMP and PTs can provide some measure of saccular function. Interestingly, the highest threshold difference (Table 2; Fig. 3) was observed for the NO and HV axes, consistent with the suggestion that saccular impairment allows distinguishing between VM and MD disease. This also explains the finding that cVEMP amplitudes, which assess saccular function, showed good sensitivity and specificity and high AUC in terms of differentiation between VM and MD patients. However, PTs may be more useful for differential diagnosis. PTs use real motion stimuli, which may lead to an advantage over cVEMP, which incorporate variability arising from across subject differences in anatomy affecting propagation of the acoustic stimulus from the speaker to the end organ. PTs also assess perceptual rather than motor function.

In contrast with cVEMP PP amplitudes, cVEMP latencies differed significantly across subject groups, being noticeably reduced in VM. As discussed above, this might reflect an increased vestibular sensitivity due to abnormal central integration of canal and otolith signals in VM. However, this result was not observed in a prior study of cVEMP in VM [38]. oVEMP provide a measure of utricular function. Our previous study [16] found an association between oVEMP n10 amplitudes and PTs (IA and NO axes), but this association was not observed in the present study, perhaps due to the higher frequency stimulus used to assess PTs (1 vs 0.5 Hz). Nor did we observe that oVEMP n10 amplitudes or latencies differed significantly across study groups, in line with previous results showing that oVEMP response does not allow separation between VM and MD [40]. These findings do not seem unexpected, because the involvement of the utricle has been reported to be less than that of the saccule in MD [15, 41]. The midline taps are relatively vigorous stimuli that may require greater utricular impairment to show abnormal responses [42]. Ocular VEMP seem to have little diagnostic relevance for VM–MD discrimination because diagnostic accuracy was the poorest of the applied tests along with the DHI questionnaire. This is also supported by prior studies showing a high interrater variability of the oVEMP method, which is very sensitive to measurement conditions [43].

Finally, analyses of PTs across patient groups are in line with previous studies. PTs along the HV axis were higher than along the IA and NO axes, probably reflecting reduced sensitivity to the predominantly vertical oscillations associated with bipedal locomotion [22, 24]. In addition, we observed a significant increase in PTs with age along all three axes, in line with prior studies [44].

In conclusion, it appears that PTs constitute a reliable technique to differentiate VM and MD, particularly for HV head motion, which depends on saccular function. VM thresholds were not reduced relative to HC, suggesting VM is not associated with a general increase in vestibular perceptual sensitivity; heightened motion sensitivity in these patients has been observed only for specific motion types [13].
